# Sex-Specific Differences in Quality and Flavor Traits of Gonads from the Sea Urchin (*Heliocidaris crassispina*): An Integrated Sensory and Metabolomic Analysis

**DOI:** 10.3390/foods15162780

**Published:** 2026-08-07

**Authors:** Dejian Su, Zirui Li, Xiuru Zhu, Qiyang Sun, Hubin Chen, Zonghe Yu

**Affiliations:** College of Marine Sciences, South China Agricultural University, Guangzhou 510642, China

**Keywords:** sea urchin, *Heliocidaris crassispina*, gonad quality, sex, flavor

## Abstract

To investigate sex-specific differences in gonad quality and flavor characteristics of the sea urchin *Heliocidaris crassispina*, female and male gonads were compared using proximate composition, colorimetry, E-tongue, E-nose, LC-MS, and HS-SPME-GC-MS. No significant sex differences were observed in wet weight, test diameter, gonadosomatic index, moisture, or crude fat, whereas female gonads showed higher crude protein content, lightness, and yellowness. E-tongue and E-nose analyses revealed distinct sex-dependent taste and odor fingerprints. Metabolomics identified 366 differential non-volatile metabolites and 53 differential volatile metabolites, mainly involving lipids, organic acids and derivatives, dipeptides, nucleotide-related compounds, hydrocarbons, terpenoids, aldehydes, ketones, and esters. Female gonads were enriched in phospholipids, lysophospholipids, dipeptides, and nucleotide precursors, whereas male gonads contained higher levels of acylcarnitines, hydroxy fatty acids, 3-carene, and hydrocarbons. These findings provide metabolomic evidence for sex-dependent flavor differences and support quality evaluation of sea urchin products.

## 1. Introduction

*Heliocidaris crassispina* is an economically valuable tropical sea urchin. It has attracted increasing attention because of its high nutritional value, desirable mouthfeel, and distinctive flavor [1,2]. Sea urchin gonads are the main edible tissues and have high nutritional value. They are therefore a key determinant of the sensory quality of sea urchin products [3]. Aquatic animals possess characteristic flavors that differ from those of terrestrial animal foods, and volatile odor-active compounds provide an important basis for their distinctive aroma and consumer preference [4]. However, studies on the flavor quality of *H. crassispina* gonads remain limited, particularly with respect to systematic characterization of taste compounds, volatile flavor compounds, and corresponding sensory attributes [5].

Sex is an important biological factor affecting the quality of edible tissues in aquatic animals. Previous studies have shown that female and male individuals may differ in proximate composition, amino acid composition, lipid composition, and fatty acid composition, thereby influencing nutritional value and sensory quality [6]. In sea urchins, the free amino acid profiles of ovaries and testes change during gonadal development, indicating that gonad flavor may be jointly regulated by sex and developmental stage [5]. Similarly, female and male mussels show different sensory attributes, volatile compound profiles, and metabolic pathway characteristics during processing, suggesting that sex-dependent differences can affect flavor formation through both flavor precursors and volatile products [7]. Therefore, comparing the quality and flavor differences between female and male *H. crassispina* gonads can help clarify the mechanisms underlying sex-dependent flavor variation.

Food flavor is determined by both volatile and non-volatile compounds. Volatile compounds, such as aldehydes, ketones, alcohols, and sulfur-containing compounds, mainly contribute to aroma [8]. Non-volatile compounds, including free amino acids, nucleotides, organic acids, sugars, and lipids, can directly affect umami, sweetness, sourness, and bitterness, and can also serve as precursors for volatile flavor compounds [9,10]. Electronic nose (E-nose) and electronic tongue (E-tongue) analyses provide rapid global fingerprints of odor and taste, respectively, whereas headspace solid-phase microextraction coupled with gas chromatography–mass spectrometry (HS-SPME-GC-MS) and liquid chromatography–mass spectrometry (LC-MS) enable molecular-level identification of volatile and non-volatile metabolites [11,12]. In recent years, the combined application of E-nose, E-tongue, GC-MS, and LC-MS has been used to analyze flavor differences in foods such as oysters, tomatoes, and apples, demonstrating the utility of this integrated approach. This integrated strategy can link sensory responses, key volatile compounds, and metabolic pathways to explain mechanisms of flavor formation [12,13,14].

In this study, proximate composition analysis, colorimetry, E-tongue, E-nose, LC-MS-based non-volatile metabolomics, and HS-SPME-GC-MS-based volatile metabolomics were combined to systematically compare basic quality, taste, and odor characteristics between female and male gonads of wild *H. crassispina*. The potential metabolic basis of sex-dependent gonad quality and flavor was further investigated, providing an integrated reference for quality evaluation, product grading, and refined utilization of sea urchin gonads.

## 2. Materials and Methods

### 2.1. Experimental Animals and Sample Collection

The *H. crassispina* specimens used in this study were collected from coastal waters of Dapeng Bay, Shenzhen (114°28′32″ E, 22°32′17″ N), on 9 July 2025. This period corresponds to the reproductive season of *H. crassispina*, when gonadal development is relatively advanced. Previous studies have shown that female and male *H. crassispina* exhibit synchronized gonadal development during this period, with both sexes reaching the mature gonadal stage (Stage IV) [15]. Twenty sea urchins were randomly selected from the collected individuals for the experiment. Wet body weight and test diameter were measured using an electronic balance and vernier calipers, respectively. The sea urchins were then dissected, and the gonads were carefully removed using clean forceps and weighed. The gonadosomatic index (GSI, %) was calculated as (GW/FBW) × 100, where GW represents gonad wet weight (g) and FBW represents the whole-body wet weight of the sea urchin (g). The isolated gonads were observed under a microscope to distinguish female and male individuals, after which they were grouped by sex. A portion of each gonad sample was immediately transferred into cryotubes and stored at −80 °C for subsequent analyses. The remaining fresh gonad samples were immediately transported to the laboratory for color analyses.

### 2.2. Proximate Composition

The proximate composition of sea urchin gonads was analyzed according to standard AOAC methods [16]. Dry matter was determined by oven drying at 60 °C for 48 h. Crude fat was extracted using a Soxtec 255 system (FOSS, Hillerød, Denmark) with diethyl ether as the solvent. Ash content was determined by combustion in a muffle furnace at 550 °C for 6 h. Nitrogen content was determined using a Kjeldahl nitrogen analyzer (Model KDN-19K, Shanghai Xianjian Instrument Co., Ltd., Shanghai, China), and crude protein content was calculated as nitrogen content multiplied by 6.25.

### 2.3. Quantification of Gonad Color Attributes

Gonad color was measured using a WR-10 colorimeter (Weifu Optoelectronics Technology, Shenzhen, China) under a D65 light source and CIE 10° standard observer conditions. Before measurement, the instrument was calibrated using a standard white plate and a black calibration cavity. Each fresh sample was measured three times. L*, a*, and b* represent lightness, redness, and yellowness, respectively. The total color differences between the measured values and each of the two standard colors, light orange-yellow (L* = 68.9, a* = 28.7, b* = 60.4) and light yellow (L* = 74.6, a* = 28.7, b* = 66.1), were defined as Δ*E_1_* and Δ*E_2_*, respectively. Δ*E* was calculated as [(ΔL*)^2^ + (Δa*)^2^ + (Δb*)^2^]^1^/^2^ [17].

### 2.4. Analysis of Electronic Nose and Electronic Tongue

An E-nose was used to evaluate odor differences between female and male *H. crassispina* gonads. Briefly, fresh gonad samples were homogenized and placed in clean 20 mL beakers, which were sealed with PE film. Headspace air in the beakers was then analyzed using a PEN 3.0 E-nose system (Airsense, Bremen, Germany). Clean air was used as the carrier gas at a flow rate of 300 mL/min. The sensor cleaning time was 120 s, and the detection phase lasted 120 s. Data collected between 70 and 75 s of detection were analyzed using the E-nose data analysis system. The system contained ten sensors with different odor sensitivities: W1C (aromatic compounds), W5S (nitrogen oxides), W3C (ammonia and aromatic compounds), W6S (hydrogen), W5C (olefins and aromatic compounds), W1S (hydrocarbons), W1W (hydrogen sulfide), W2S (alcohols and partially aromatic compounds), W2W (aromatic compounds and organic sulfides), and W3S (alkanes and methane).

An E-tongue was used to evaluate taste differences between female and male *H. crassispina* gonads. Gonad samples (1.2 g) were mixed with ultrapure water at a ratio of 1:5 and homogenized for 1 min. The homogenate was centrifuged at 4 °C. The supernatant (4 mL) was diluted to 80 mL with ultrapure water before analysis. An Alpha-Astree E-tongue system (Alpha MOS, Toulouse, France) was used, and each sample was analyzed for 120 s after signal stabilization. The system contained seven sensors corresponding to different taste attributes: NMS (umami), CTS (saltiness), AHS (sourness), SCS (bitterness), ANS (sweetness), PKS (richness/complexity), and CPS (comprehensive aftertaste).

### 2.5. Non-Volatile Metabolite Analysis by LC-MS

Samples stored at −80 °C were transferred to ice and subsequently ground in liquid nitrogen. Approximately 20 mg of each powdered sample was extracted with 400 μL of methanol/water (7/3, *v*/*v*) containing the following internal standards: (S)-4-fluorophenylglycine, D-luciferin, 2-amino-3-(2-chlorophenyl)propanoic acid, LPC(16:0)-d31, palmitic acid-d31, benzoic acid-d5, hexanoic acid-d11, and tridecanoic acid-d2. After vortexing at 1500 rpm for 5 min and incubation on ice for 15 min, the mixture was centrifuged at 12,000 rpm for 10 min at 4 °C. The supernatant was transferred to a new tube, precipitated at −20 °C for 30 min, and centrifuged again at 12,000 rpm for 3 min at 4 °C. The clarified supernatant was used for LC-MS analysis. A pooled quality-control (QC) sample was prepared by combining the sample extracts, and three replicate QC analyses (QC01–QC03) were included in the analytical sequence to monitor analytical repeatability and stability. LC-MS analysis was performed using a Thermo Scientific Vanquish UPLC system coupled to a Q Exactive HF-X mass spectrometer (Thermo Fisher Scientific, Bremen, Germany). Separation was carried out on a Waters ACQUITY Premier HSS T3 column (1.8 μm, 2.1 × 100 mm) with 0.1% formic acid in water as mobile phase A and 0.1% formic acid in acetonitrile as mobile phase B. The A/B gradient was programmed as follows: 95:5 at 0 min, 80:20 at 2 min, 40:60 at 5 min, 1:99 at 6 min, maintained at 1:99 until 7.5 min, returned to 95:5 at 7.6 min, and maintained at 95:5 until 10 min. The column temperature was 40 °C, the flow rate was 0.4 mL/min, and the injection volume was 3 μL. Samples were analyzed in both positive and negative ESI modes. The spray voltages were 3.8 kV and 3.4 kV in positive and negative modes, respectively. The sheath gas and auxiliary gas flow rates were set to 60 and 20 arbitrary units, respectively. The ion transfer tube temperature was maintained at 320 °C in both positive and negative ionization modes. Full-scan MS data were acquired over an *m*/*z* range of 70–1000 at a resolution of 60,000, with an automatic gain control (AGC) target of 3 × 10^6^ and an RF lens setting of 50. MS/MS spectra were acquired over an *m*/*z* range of 70–1000 at a resolution of 15,000, with an AGC target of 1 × 10^5^, stepped collision energies of 30, 40, and 50 eV, a signal intensity threshold of 1 × 10^6^, a Top N setting of 10, and a dynamic exclusion setting of 3.

Raw data were converted to mzML format using ProteoWizard version 3.0.7414 and processed using XCMS version 3.2 for peak extraction, alignment, and retention time correction. Peaks with missing values in more than 50% of the samples within a group were removed. For the remaining data, missing values were imputed using the K-nearest neighbors method implemented in the impute package (version 1.56.0) when the proportion of missing values was less than 50%; otherwise, they were replaced with one-fifth of the minimum value. Peak-area drift was corrected using support vector regression. Metabolites were annotated using the MetWare in-house database, public databases, and prediction databases. Metabolites with an annotation score > 0.5 and a QC coefficient of variation < 0.3 were retained for subsequent analysis.

### 2.6. Volatile Metabolite Analysis by HS-SPME-GC-MS

Before analysis, each sample was ground under liquid nitrogen. Approximately 0.2 g of powder was transferred into a 20 mL headspace vial, followed by the addition of 0.2 g NaCl and 20 μL of 3-hexanone-2,2,4,4-d4 internal standard solution (10 μg/mL). The vial was immediately sealed for HS-SPME-GC-MS analysis. A pooled quality-control (QC) sample was prepared by combining the samples and processed using the same procedure, and three replicate QC analyses (QC01–QC03) were included in the analytical sequence to monitor analytical repeatability and stability. Volatile compounds were extracted using a 120 μm DVB/CWR/PDMS SPME Arrow fiber at 60 °C for 15 min after equilibration at 60 °C for 5 min, and then desorbed at 250 °C for 5 min. GC-MS analysis was performed using an Agilent 8890 gas chromatograph coupled to an Agilent 7000D mass spectrometer (Agilent Technologies, Santa Clara, CA, USA) equipped with a DB-5MS capillary column (30 m × 0.25 mm, 0.25 μm). Helium was used as the carrier gas at a constant flow rate of 1.2 mL/min. The oven temperature was held at 40 °C for 3.5 min, increased to 100 °C at 10 °C/min, then to 180 °C at 7 °C/min, and finally to 280 °C at 25 °C/min, where it was held for 5 min. Mass spectra were acquired in EI mode at 70 eV using selected ion monitoring.

Volatile compounds were identified based on retention time, retention index, characteristic ions, literature information, partial authentic standards, and the MetWare in-house database. Relative quantification was performed using quantitative ion peak areas normalized to the internal standard, and compounds with a QC coefficient of variation < 0.5 were retained.

Aroma descriptions were obtained from online databases including The Good Scents Company, Perflavory, Odour.org.uk, Food Flavor Lab, and the relevant literature. Relative odor activity values (rOAVs) were calculated as rOAVi = Ci/Ti, where Ci represents relative content and Ti represents the reported odor threshold [12]. Compounds with rOAV ≥ 1 were regarded as candidate aroma-active compounds. Because volatile compounds were semi-quantified using internal-standard-normalized data rather than absolute concentrations, rOAVs were used only for preliminary screening of candidate aroma-active compounds and should not be interpreted as conventional OAVs calculated from absolute concentration data.

### 2.7. Statistical Analysis and Data Processing

E-tongue, E-nose, non-volatile metabolomics, and volatile metabolomics data were analyzed and visualized using R version 4.1.2. The stats package was used for PCA and plotting after log2 transformation and zero-centering. MetaboAnalystR version 1.0.1 was used for OPLS-DA after log2 transformation and zero-centering, and model robustness was evaluated using 200 permutation tests. ComplexHeatmap version 2.9.4 was used for hierarchical heatmap analysis and visualization after unit variance scaling. The WGCNA package (version 1.69) was used to calculate correlation coefficients between E-tongue responses and non-volatile metabolites, and between E-nose responses and volatile metabolites, and the corrplot package (version 0.92) was used for visualization. Identified metabolites were annotated using the KEGG Compound database and then mapped to the KEGG Pathway database.

For E-tongue, E-nose, LC-MS, and HS-SPME-GC-MS analyses, three independent gonad samples from each sex were selected as biological replicates, whereas biometric parameters, color, and proximate composition were analyzed using all available individuals unless otherwise specified. All statistical analyses were conducted using JMP Pro 16 (SAS Institute Inc., Cary, NC, USA). Biometric parameters, GSI, proximate composition, and color were compared between female and male sea urchins using *t*-tests. Normality and homogeneity of variance were assessed before analysis. Results are presented as mean ± standard deviation.

## 3. Results and Discussion

### 3.1. Fundamental Characteristics of Gonad Samples

#### 3.1.1. Biometric Parameters and Gonadosomatic Index

Microscopic examination of the gonads showed that the 20 sea urchins used in this study comprised 12 males and 8 females. Wet weight and test diameter were 62.61 ± 21.12 g and 50.16 ± 4.10 mm in males, respectively, and 70.89 ± 20.92 g and 52.63 ± 6.80 mm in females, respectively (Table 1). Wet weight and test diameter did not differ significantly between sexes (*t*-test, *p* = 0.400 and *p* = 0.322, respectively). Similarly, GSI did not differ significantly between males and females, with values of 5.88 ± 2.20 and 5.98 ± 1.14, respectively (*t*-test, *p* = 0.912). These results indicate that, under the present experimental conditions, sex was not a determining factor for gonad size in *H. crassispina*. This finding is consistent with previous studies on *Strongylocentrotus intermedius*, *Paracentrotus lividus*, and *Evechinus chloroticus*, in which gonad wet weight and GSI did not necessarily differ significantly between sexes [18,19,20].

#### 3.1.2. Proximate Composition of Sea Urchin Gonads

In male and female *H. crassispina* gonads, moisture ranged from 67.09% to 67.64%, and crude fat ranged from 29.51% to 29.73%. Neither parameter differed significantly between sexes (*t*-test, both *p* > 0.05; Table 1). Crude ash content was significantly higher in male gonads (8.90 ± 0.72%) than in female gonads (5.14 ± 0.22%; *t*-test, *p* = 0.001). In contrast, crude protein content was significantly higher in female gonads (42.97 ± 0.07%) than in male gonads (36.18 ± 0.05%; *t*-test, *p* < 0.001).

The higher protein content in female gonads may be related to nutrient accumulation during ovarian development. Previous studies on sea urchin gonads have shown that reproductive stage affects nutritional composition and that sweet and umami amino acids, such as glycine and glutamic acid, may increase more markedly in female gonads during development [5,19]. Therefore, the higher crude protein and lower relative ash contents in female *H. crassispina* gonads may partly reflect sex-dependent differences in gonadal development and nutrient allocation.

#### 3.1.3. Colorimetric Analysis

Previous studies on sensory acceptance have shown a strong association between color and consumer sensory descriptions. Female gonads are often associated with orange color and attractive appearance, whereas male gonads are more frequently associated with brown, milky white, or less attractive appearances [21]. In the present study, colorimetric analysis showed that a* was significantly higher in male gonads than in female gonads (20.35 ± 1.30 vs. 17.88 ± 2.12; *t*-test, *p* = 0.006), whereas L* and b* were significantly lower in male gonads (*t*-test, both *p* < 0.001; Table 2). Because high-quality sea urchin gonads are generally expected to show a bright yellow to orange-yellow color rather than a simply reddish or dark tone, integrated color difference values may better reflect commercial value than a single chromaticity parameter [17,22]. Compared with two preferred standard gonad colors, female gonads had Δ*E*_1_ and Δ*E*_2_ values of 23.21 ± 1.08 and 30.43 ± 1.33, respectively, which were significantly lower than the corresponding values in male gonads (35.33 ± 2.02 and 43.14 ± 2.01; *t*-test, both *p* < 0.001). Although male gonads showed higher redness, their higher Δ*E*_1_ and Δ*E*_2_ values indicate greater deviation from consumer-preferred standard colors. Female gonads, with higher lightness and yellowness, showed an overall color closer to the preferred yellow to orange-yellow appearance.

Sex-related color differences in sea urchin gonads may be associated with differences in carotenoid deposition, nutrient storage status, and gonadal tissue structure. Previous studies suggest that female gonads often accumulate more carotenoids, especially pigments related to yellow-orange coloration, while differences in gametogenic stage and tissue composition can further affect pigment distribution and visual appearance [19,21,22]. Therefore, the brighter and more yellow-orange color of female *H. crassispina* gonads may partly reflect sex-dependent pigment accumulation and gonadal development characteristics.

### 3.2. Electronic Tongue and Electronic Nose Analyses

#### 3.2.1. Electronic Tongue Analysis

**Figure 1:**
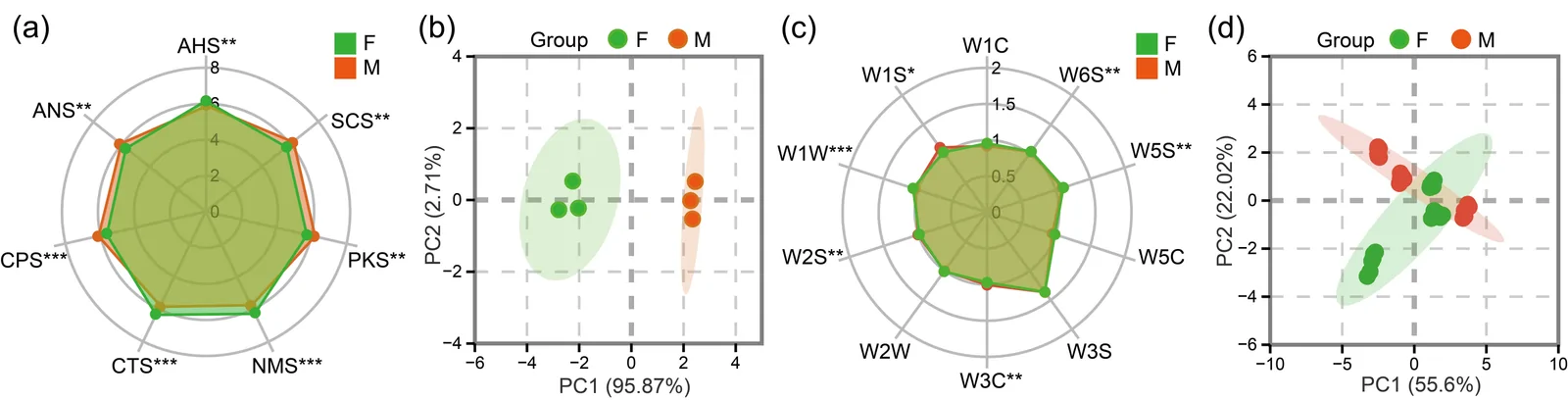
Electronic tongue (E-tongue) and electronic nose (E-nose) analyses of male and female *Heliocidaris crassispina* gonads. (**a**,**b**) Radar chart and PCA plot of the E-tongue. (**c**,**d**) Radar chart and PCA plot of the E-nose. Asterisks indicate significant differences (* *p* < 0.05, ** *p* < 0.01, *** *p* < 0.001).

The taste profiles of female and male *H. crassispina* gonads were analyzed using the Alpha-Astree E-tongue system. All sensor responses differed significantly between sexes (*t*-test, all *p* < 0.01; Figure 1a,b), indicating that sex affected the overall taste fingerprint of sea urchin gonads. Female gonads showed higher responses for NMS, CTS, and AHS, which are mainly related to umami, salty/umami taste, and sourness, whereas male gonads showed stronger responses for SCS, ANS, PKS, and CPS, corresponding to bitterness, sweetness, and comprehensive taste responses.

E-tongue signals reflect integrated responses from multiple water-soluble taste compounds rather than changes in a single compound. Previous studies have shown that taste differences in sea urchin gonads are closely associated with free amino acids, nucleotides, organic acids, and their interactions, especially in relation to umami and bitterness [23]. Therefore, the stronger umami and salty/umami responses in female gonads may be related to their higher crude protein content and the accumulation of protein-derived amino acids, small peptides, or nucleotide-related compounds. In contrast, the stronger sweetness, bitterness, and comprehensive responses in male gonads may reflect differences in sweet amino acids, nitrogen-containing small molecules, organic acids, or other soluble metabolites [14].

#### 3.2.2. Electronic Nose Analysis

The E-nose does not identify individual volatile compounds, but provides an integrated response to headspace volatiles and is therefore suitable for comparing odor fingerprints [24]. In this study, six of the ten sensor responses differed significantly between female and male *H. crassispina* gonads, including W5S, W3C, W6S, W1S, W1W, and W2S (*t*-test, *p* < 0.05; Figure 1c), whereas W1C, W5C, W2W, and W3S showed no significant differences (*p* > 0.05). PCA also showed partial separation between sexes, although some overlap remained (Figure 1d). These results indicate that sex affected the volatile odor fingerprint of sea urchin gonads, but the overall odor profiles were not completely distinct.

Female gonads showed higher responses for W5S, W6S, and W1W, while male gonads showed higher responses for W3C, W1S, and W2S. This pattern suggests sex-related differences in specific volatile response characteristics rather than a uniformly stronger odor profile in either sex. Considering that lipid oxidation, amino acid degradation, and nitrogen- or sulfur-containing compounds can affect volatile flavor, these sensor differences may reflect variation in the release of different classes of headspace volatiles [25]. Previous studies also showed that sea urchin gonad quality is closely related to pleasant odor compounds such as butyl acetate and benzaldehyde, as well as off-odors from sulfur-containing compounds, with low sulfur odor, high umami, and low bitterness being important characteristics of high-quality gonads [23].

### 3.3. Analysis of Differential Non-Volatile Metabolites by LC-MS

#### 3.3.1. Non-Volatile Metabolomics Analysis

**Figure 2:**
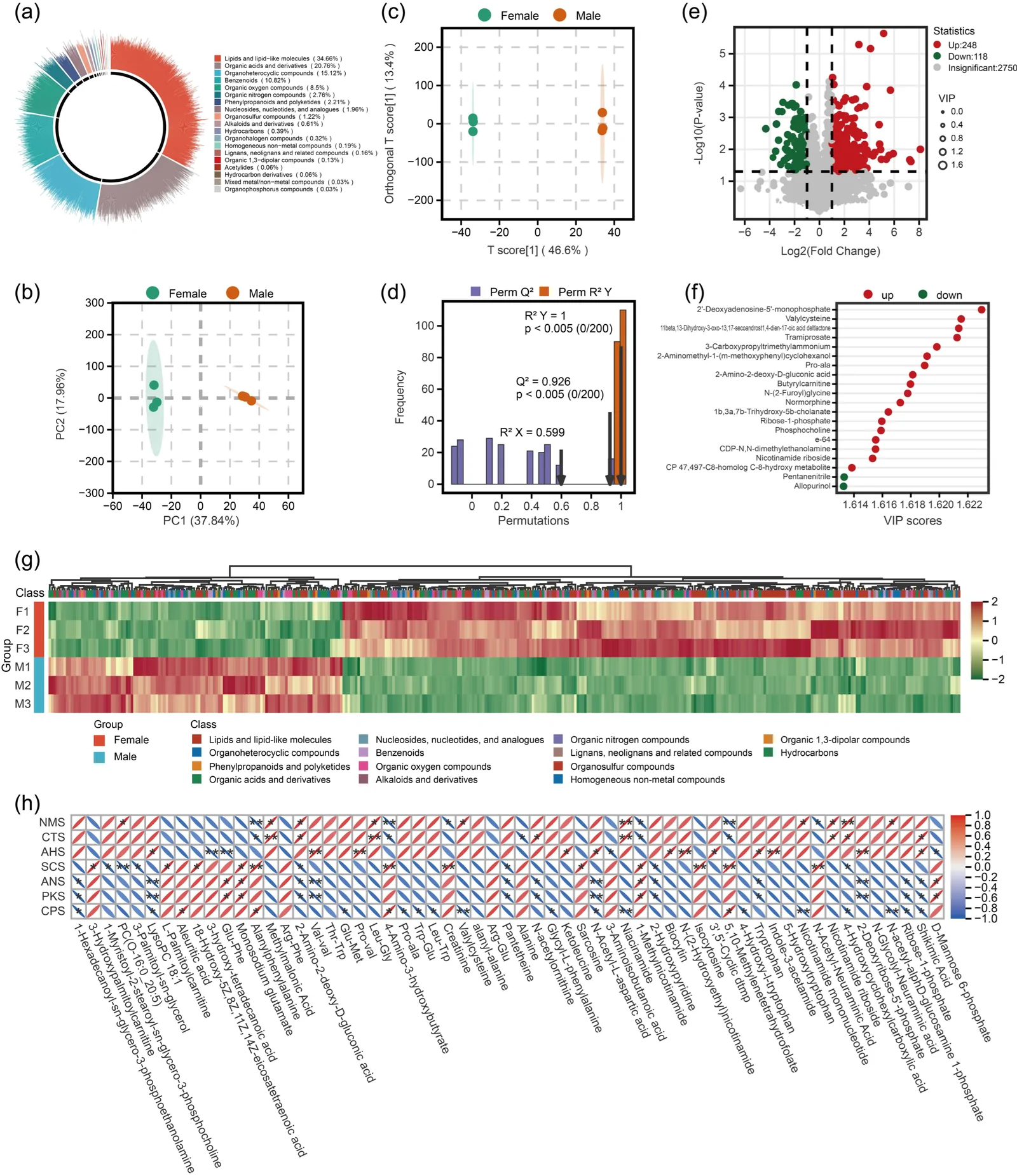
Non-volatile metabolomics analysis with males as the control group. (**a**) Ring chart of non-volatile metabolite composition; (**b**) PCA plot; (**c**) OPLS-DA score plot; (**d**) model validation plot; (**e**) volcano plot of differentially regulated non-volatile metabolites; (**f**) VIP analysis of the top 20 metabolites; (**g**) heatmap clustering of differential metabolites; and (**h**) correlation analysis between differential non-volatile metabolites and electronic tongue responses. Ellipses represent Spearman rank correlation coefficients. Asterisks indicate significant correlations (* *p* < 0.05, ** *p* < 0.01). Red indicates positive correlation (*r* > 0.3), with *r* > 0.7 representing a strong positive correlation. Blue indicates negative correlation (*r* < −0.3), with *r* < −0.7 representing strong negative correlation.

To further investigate taste differences between the two sexes of *H. crassispina*, LC-MS-based non-volatile metabolomics was performed on gonad samples. A total of 3660 non-volatile metabolites were detected, of which 3116 were annotated, including 1080 lipids and lipid-like molecules (34.66%), 647 organic acids and derivatives (20.76%), 471 organoheterocyclic compounds (15.12%), 337 benzenoids (10.82%), 265 organic oxygen compounds (8.50%), 86 organic nitrogen compounds (2.76%), 69 phenylpropanoids and polyketides (2.21%), 61 nucleosides, nucleotides, and analogues (1.96%), 38 organosulfur compounds (1.22%), and 62 others (1.99%) (Figure 2a, Table S1). The unsupervised PCA plot showed clear separation between female and male samples in principal component space, with good clustering of samples within each group (Figure 2b). These results indicate that sex had a clear effect on the non-volatile metabolite profile of *H. crassispina* gonads and that the biological replicates showed good reproducibility.

#### 3.3.2. Differential Analysis of the Non-Volatile Metabolome

OPLS-DA is an effective approach for screening differentially abundant metabolites, and variable importance in projection (VIP) values provide a basis for further analysis. OPLS-DA was therefore performed to examine differences between the two groups. The results showed clear separation between female and male gonads (Figure 2c). The R^2^X, R^2^Y, and Q^2^ values were 0.599, 1.000, and 0.926, respectively, indicating good explanatory and predictive performance of the model (Figure 2d). VIP scores derived from the OPLS-DA model were used to identify differential metabolites (VIP ≥ 1). Volcano plots were generated using the criteria VIP ≥ 1, fold change ≥ 2 or ≤0.5, and *p* < 0.05 (Figure 2e,f, Table S2). A total of 366 differential metabolites were identified, including 248 metabolites upregulated and 118 metabolites downregulated in female gonads relative to male gonads. These metabolites included 132 lipids and lipid-like molecules (36.07%), 89 organic acids and derivatives (24.32%), 45 organoheterocyclic compounds (12.30%), 32 organic oxygen compounds (8.74%), 28 benzenoids (7.65%), and 40 others (10.92%) (Figure 2g). These metabolites may represent important molecular contributors to sex-dependent taste differences in sea urchin gonads. Subsequently, 59 key differential metabolites were selected from the 366 differential metabolites for Spearman correlation analysis with E-tongue responses, and the results were visualized as a heatmap (Figure 2h, Table S3).

#### 3.3.3. Lipids and Lipid-like Molecules

Lipids and lipid-like molecules are not classical water-soluble taste compounds, but they can influence overall flavor through texture, oral lubrication, fat-related perception, aroma retention, and the formation of lipid-derived volatiles [26,27]. Lipid hydrolysis and oxidation may generate aldehydes, ketones, alcohols, acids, and other aroma-active compounds, thereby affecting aroma complexity and flavor harmony [28,29].

In female gonads, phospholipids, lysophospholipids, and glycerolipids such as 1-Hexadecanoyl-sn-glycero-3-phosphoethanolamine, 1-Myristoyl-2-stearoyl-sn-glycero-3-phosphocholine, PC(O-16:0_20:5), 3-Palmitoyl-sn-glycerol, and LysoPC 18:1 showed higher relative abundances (Figure 2g, Table S3). This result may reflect more pronounced membrane lipid remodeling, oocyte structural expansion, and lipid storage in female gonads [30]. Sea urchin gonads function as both gametogenic tissues and nutrient storage tissues. During gametogenesis, nutrients stored in nutritive phagocytes can be mobilized and transferred to germ cells, while female germ cell development is usually accompanied by oocyte enlargement and nutrient accumulation [19]. Previous studies have also shown that the nutritional composition of sea urchin gonads is affected by sex, season, and gametogenic cycle, and that polyunsaturated fatty acids can show a stronger tendency to accumulate in female gonads [19]. Therefore, the higher levels of phospholipids and lysophospholipids in female gonads may be associated with oocyte development, membrane renewal, and lipid nutrient accumulation. These lipid compounds may not directly produce umami or sweetness, but they can enhance taste fullness and harmony by changing the gonad matrix structure, lipid phase proportion, flavor release rate, oral lubrication, and persistent perception [31,32]. Polar lipids and phospholipids can also serve as important precursors for lipid oxidation and volatile flavor formation, thereby affecting overall flavor performance [29,33].

In contrast, long-chain acylcarnitines, hydroxy fatty acids, and oxidized lipid-related metabolites, including L-Palmitoylcarnitine, 3-Hydroxypalmitoylcarnitine, 18-Hydroxy-5Z,8Z,11Z,14Z-eicosatetraenoic acid, 3-hydroxy-tetradecanoic acid, and Aleuritic acid, were more abundant in male gonads. Acylcarnitines are closely related to fatty acid transport and β-oxidation, suggesting stronger fatty acid catabolism and energy supply in male gonads [6,34]. These lipid degradation- or oxidation-related metabolites may not directly affect taste attributes. Instead, they may indirectly influence E-tongue responses by altering soluble small-molecule composition, lipid oxidation level, fatty taste background, and aftertaste characteristics.

#### 3.3.4. Organic Acids and Derivatives

The differential organic acids and derivatives mainly included free amino acids, N-acetylated amino acids, hydroxy/keto acids, dipeptides, and other nitrogen-containing small organic molecules. These compounds are important non-volatile taste components in aquatic products and may contribute to umami, sweetness, sourness, bitterness, salty/umami taste, and aftertaste [31,35]. In sea urchin gonads, free amino acids and nucleotides are considered key taste-related compounds, with Gly and Ala commonly associated with sweetness, Glu with umami, and Arg, Lys, His, and Val with bitterness or complex aftertaste [36,37].

Female gonads showed higher relative abundances of several N-acetylated amino acids and dipeptides, whereas glutamate, alanine, sarcosine, and several Phe- or Arg-containing dipeptides were more abundant in male gonads (Figure 2g, Table S3). At the compound-class level, these differences are consistent with previously reported sex-related variation in water-soluble taste-related metabolites in sea urchin gonads [5,20]. However, the sensory contribution of an individual amino acid derivative or peptide cannot be assigned from untargeted relative-abundance data alone. The observed E-tongue separation is therefore more appropriately interpreted as an integrated response to differences in multiple soluble metabolites.

#### 3.3.5. Organoheterocyclic Compounds

Organoheterocyclic compounds mainly included pyridines, pyrimidines, indoles, pteridine/folate-related compounds, and their derivatives. These nitrogen-containing cyclic compounds are commonly associated with nucleotides, coenzymes, vitamins, and other metabolic regulatory molecules [38]. In female gonads, Niacinamide, 2-Hydroxypyridine, Biocytin, N-(2-Hydroxyethyl)nicotinamide, 3′,5′-Cyclic dTMP, Tryptophan, 4-Hydroxy-L-tryptophan, 5-Hydroxytryptophan, and Indole-3-acetamide showed higher relative abundances (Figure 2g, Table S3). These metabolites indicate that female gonads may have relatively higher levels of nicotinamide-related compounds, nucleotide-related metabolites, and tryptophan derivatives. Among them, tryptophan and some hydrophobic amino acid-related metabolites may contribute to bitterness or aftertaste complexity, although the final taste perception is likely determined by interactions among amino acids, nucleotides, organic acids, and other water-soluble compounds [5,36,39].

In contrast, 1-Methylnicotinamide, Isocytosine, and 5,10-Methylenetetrahydrofolate were more abundant in male gonads. These compounds suggest sex-related differences in nitrogen-containing heterocyclic metabolite profiles, especially those associated with nicotinamide-, pyrimidine-, and folate-related compounds [40].

#### 3.3.6. Organic Oxygen Compounds

The differential organic oxygen compounds mainly included sugar phosphates, ribose-related metabolites, amino sugars, sialic acid compounds, and NAD^+^ salvage-related precursors [41]. In female gonads, Nicotinamide mononucleotide, Nicotinamide riboside, 2-Deoxyribose-5′-phosphate, Ribose-1-phosphate, N-acetyl-alpha-D-glucosamine 1-phosphate, 4-Hydroxycyclohexylcarboxylic acid, Shikimic acid, and N-Glycolyl-neuraminic acid showed higher relative abundances (Figure 2g, Table S3). These metabolites suggest that female gonads may be characterized by relatively higher levels of coenzyme-related precursors, ribose/sugar phosphate compounds, and glycan-related metabolites [42].

In contrast, N-Acetyl-neuraminic acid and D-Mannose 6-phosphate were more abundant in male gonads. As representative sialic acid and sugar phosphate compounds, respectively, their enrichment indicates sex-related differences in carbohydrate- and glycoconjugate-related metabolite profiles [43,44]. Notably, the higher abundance of Shikimic acid in female gonads should be interpreted cautiously, as the shikimate pathway is generally absent in animals and may reflect exogenous inputs, such as dietary algal sources [45]. Overall, these relative-abundance differences should be interpreted as sex-associated metabolic markers rather than direct evidence of differences in glycan synthesis, coenzyme turnover, or energy metabolism.

### 3.4. Analysis of Differential Volatile Flavor Compounds by HS-SPME-GC-MS

#### 3.4.1. Volatile Metabolomics Analysis

**Figure 3:**
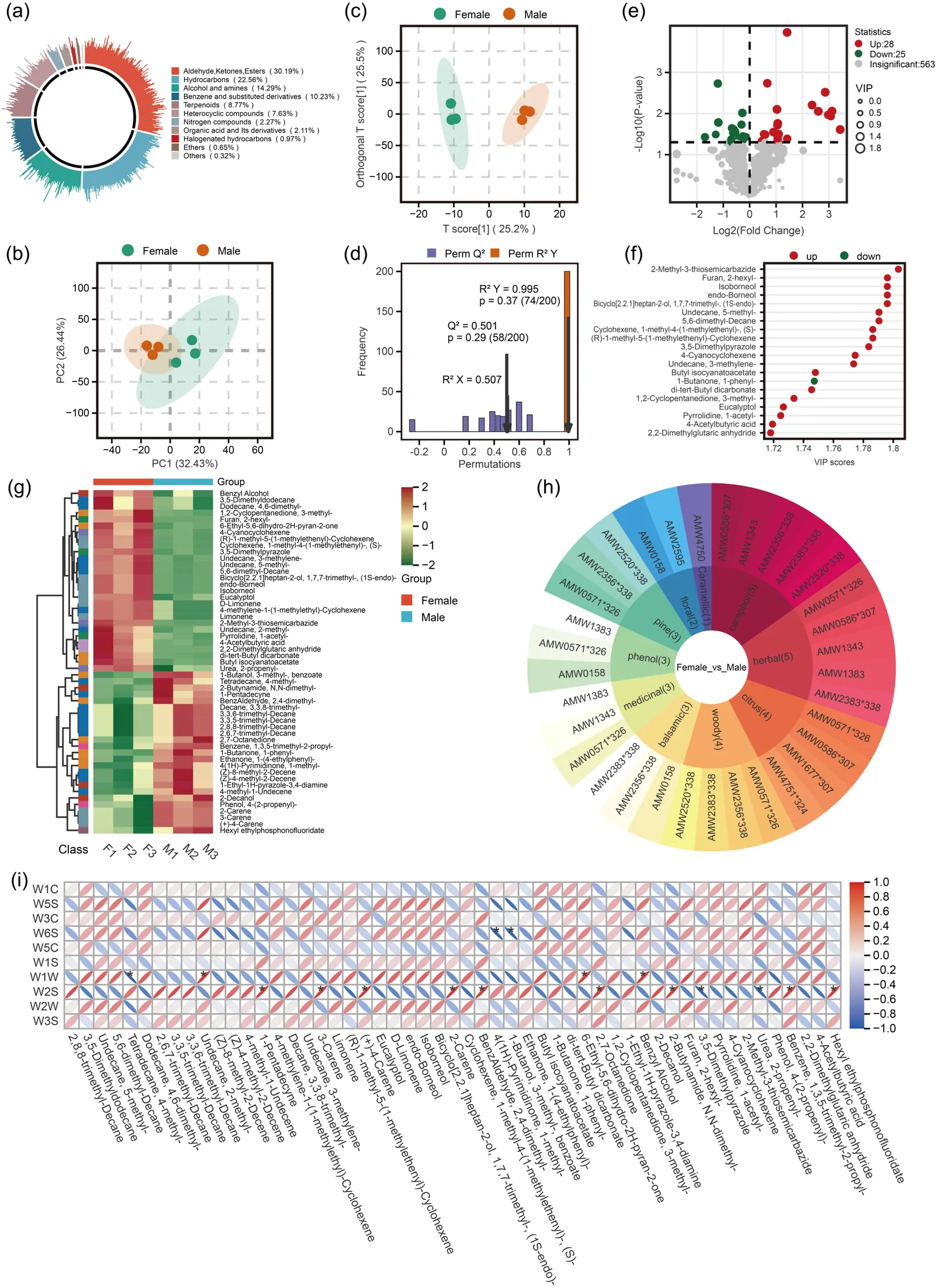
Volatile metabolomics analysis with males as the control group. (**a**) Ring chart of volatile metabolite composition; (**b**) PCA plot; (**c**) OPLS-DA score plot; (**d**) model validation plot; (**e**) volcano plot of differentially regulated volatile metabolites; (**f**) VIP analysis of the top 20 metabolites; (**g**) heatmap clustering of differentially abundant metabolites; (**h**) flavor wheel of differentially abundant metabolites showing the top 10 sensory descriptors with the highest number of annotations; and (**i**) correlation analysis between differential volatile compounds and electronic nose sensor responses. Ellipses represent Spearman rank correlation coefficients. Asterisks indicate significant correlations (*p* < 0.05). Red indicates positive correlation (*r* > 0.3), with *r* > 0.7 representing a strong positive correlation. Blue indicates negative correlation (*r* < −0.3), with *r* < −0.7 representing strong negative correlation.

To further investigate odor differences between the two sexes of *H. crassispina*, HS-SPME-GC-MS-based volatile metabolomics was performed on gonad samples. A total of 621 volatile metabolites were detected, of which 616 were identified, including 186 aldehydes, ketones, and esters (30.19%), 139 hydrocarbons (22.56%), 88 alcohols and amines (14.29%), 63 benzenes and substituted derivatives (10.23%), 54 terpenoids (8.77%), 47 heterocyclic compounds (7.63%), 14 nitrogen compounds (2.27%), 13 organic acids and derivatives (2.11%), 6 halogenated hydrocarbons (0.97%), 4 ethers (0.65%), and 2 others (0.32%) (Figure 3a, Table S4). The unsupervised PCA plot showed separation between male and female gonads based on volatile metabolite profiles, with the first two principal components explaining 50.7% of the total variance (Figure 3b).

#### 3.4.2. Differential Analysis of the Volatile Metabolome

Subsequent OPLS-DA showed clear separation between the two groups (Figure 3c). The R^2^X, R^2^Y, and Q^2^ values were 0.507, 0.995, and 0.501, respectively, indicating that the OPLS-DA model showed acceptable discrimination between female and male gonads, although the moderate Q^2^ value suggests that the model should be interpreted cautiously (Figure 3d). VIP scores derived from the OPLS-DA model were used to identify differential metabolites (VIP ≥ 1), and volcano plots were generated using the criteria VIP ≥ 1 and *p* < 0.05 (Figure 3e,f, Table S4). A total of 53 differential metabolites were identified, including 28 metabolites upregulated and 25 metabolites downregulated in female gonads relative to male gonads. These metabolites included 17 hydrocarbons (32.08%), 11 terpenoids (20.75%), 10 aldehydes, ketones, and esters (18.87%), 4 alcohols and amines (7.55%), 3 heterocyclic compounds (5.66%), 3 nitrogen compounds (5.66%), 2 organic acids and derivatives (3.77%), 2 benzenes and substituted derivatives (3.77%), and 1 other compound (1.89%) (Figure 3g, Table S5). These differential metabolites may be important contributors to sex-dependent odor differences in sea urchin gonads. The flavor wheel summarized the odor descriptors enriched among the differential metabolites (Figure 3h). The top ten flavor descriptors were camphor, herbal, citrus, woody, balsamic, medicinal, phenol, pine, floral, and caramellic. Differences in these odor attributes jointly shaped the odor profiles of female and male gonads. Spearman correlation analysis was then performed between the 53 differential volatile metabolites and E-nose responses, and the results were visualized as a heatmap (Figure 3i, Table S6).

#### 3.4.3. Hydrocarbons

Hydrocarbons accounted for the largest proportion of the 53 differential volatile metabolites and included various branched alkanes, alkenes, and alkynes, such as Undecane, 2-methyl-, 3,5-Dimethyldodecane, Undecane,5-methyl-, Dodecane,4,6-dimethyl-, (Z)-8-methyl-2-Decene, and 1-Pentadecyne (Figure 3g, Table S6). Because hydrocarbons generally have relatively high odor thresholds, they are more appropriately interpreted as sex-associated chemical markers than as direct odorants. Their differential abundance may be consistent with variation in lipid-derived or exogenous volatile pools; however, the present data do not distinguish their sources or establish sex-dependent differences in lipid oxidation.

#### 3.4.4. Terpenoids

Terpenoids were a noteworthy class of differential volatile compounds in this study because monoterpenes and oxygenated monoterpenes usually have clear odor attributes and can modify volatile flavor in foods and seafood [46,47]. In female gonads, Limonene, D-Limonene, Eucalyptol, endo-Borneol, Isoborneol, and Bicyclo[2.2.1]heptan-2-ol,1,7,7-trimethyl-,(1S-endo)- showed higher relative abundances (Figure 3g, Table S6). In contrast, carene isomers such as 3-carene, (+)-4-carene, and 2-carene were more abundant in male gonads. Terpenoids are commonly associated with odor attributes such as citrus, herbal, camphor, pine, woody, and balsamic. Limonene-related compounds are usually associated with citrus, herbal, terpene, and camphor notes. Previous volatile organic compounds studies on sea urchin gonads also found that limonene could be perceived by GC-sniffing and may contribute to desirable odor characteristics in sea urchin gonads [48]. Eucalyptol and Isoborneol are associated with eucalyptus, herbal, camphor, medicinal, balsamic, and woody notes. However, most terpenoids had rOAV values below 1, ranging from 0.01 to 0.18, suggesting that they may function mainly as background odor modifiers.

In contrast, 3-carene had an rOAV of 9.3, indicating that it may be an important candidate odorant contributing to flavor differences between female and male gonads. 3-carene is mainly associated with citrus, terpenic, herbal, pine, solvent, resinous, phenol, cypress, medicinal, and woody notes. It was significantly positively correlated with W2S (*r* = 0.87, *p* = 0.024), suggesting that 3-carene may be associated with sex-related variation in specific E-nose sensor responses. 3-carene has been identified as an abundant aroma metabolite in fruits such as mango and pomegranate and is considered a key volatile metabolite during the ripening of mango and *Alpinia hainanensis* (Zingiberaceae) [49,50,51]. In this study, 3-carene may be one of the important metabolites affecting sex-dependent gonad flavor differences in sea urchins. Terpenoids in sea urchin gonads may also be partly influenced by dietary algae, environmental sources, or in vivo accumulation processes [48].

#### 3.4.5. Aldehydes, Ketones, and Esters

Aldehydes, ketones, and esters usually have relatively low thresholds and may contribute more directly to odor. Studies on aquatic animal flavor generally consider aldehydes, ketones, and alcohols to be important volatile flavor components [9]. Aldehydes are mostly generated through fatty acid oxidation and Strecker degradation, and their low odor thresholds allow them to strongly influence fishy, fatty, grassy, or fruity notes [52]. In this study, 1,2-Cyclopentanedione,3-methyl- was more abundant in female gonads, whereas several aromatic aldehydes and ketones were more abundant in male gonads (Figure 3g, Table S6). Although aromatic aldehydes, including benzaldehyde, have previously been associated with sea urchin aroma [23,53], specific sensory notes cannot be assigned to the differential compounds identified here without GC–olfactometry and matrix-specific absolute OAVs. These results therefore support a sex-associated difference in the carbonyl profile rather than stronger predefined odor notes in either sex.

#### 3.4.6. Alcohols and Amines

Alcohols and amines may also contribute to odor differences between female and male sea urchin gonads. Alcohols are common volatile components in aquatic products and animal-derived foods, whereas some volatile amines, especially lower amines such as trimethylamine, are associated with fishy or spoilage odors in aquatic products [9]. Benzyl alcohol was more abundant in female gonads, whereas 2-decanol and several nitrogen-containing alcohols or amines were more abundant in male gonads (Figure 3g, Table S6). Because benzyl alcohol had an rOAV of only 0.1 and most alcohols have relatively high odor thresholds, these compounds are considered components of the differential volatile profile, but their direct sensory contributions cannot be resolved from the present data. Nevertheless, they can still affect the overall odor profile when present at high relative abundance or in synergy with other volatiles [52].

#### 3.4.7. Heterocyclic Compounds and Nitrogen Compounds

Several heterocyclic and nitrogen-containing compounds also differed between female and male gonads (Figure 3g, Table S6). Although these chemical classes can include low-threshold odorants, the sensory contributions of the individual compounds detected here were not experimentally verified. In particular, Urea, 2-propenyl- showed a high screening rOAV, but no well-defined odor descriptor or matrix-specific threshold was available. It was therefore not considered a confirmed key odorant and requires validation using an authentic standard, absolute quantification, and GC–olfactometry.

### 3.5. Integrated Pathway Analysis Reveals Potential Mechanisms Underlying Sex-Dependent Taste and Odor Formation

**Figure 4:**
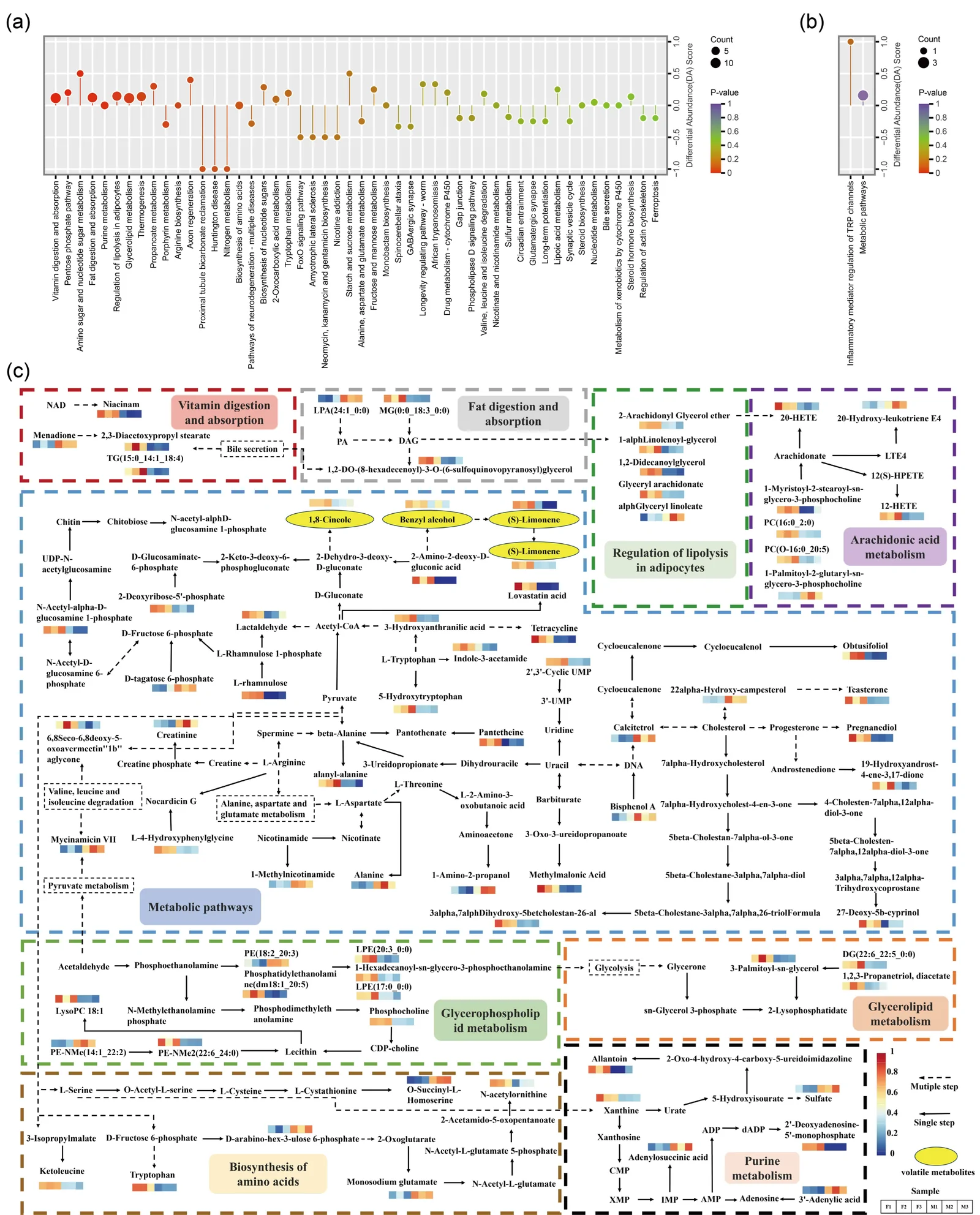
Sex-dependent taste and flavor metabolic network in *Heliocidaris crassispina* gonads, with males used as the control group. (**a**,**b**) DA-score lollipop plots showing pathway enrichment of non-volatile and volatile metabolites, respectively. (**c**) Proposed metabolite interaction network potentially contributing to taste and flavor variation. The heatmap shows metabolite intensity, with red indicating upregulation and blue indicating downregulation. F and M denote female and male samples, respectively. Solid arrows indicate direct metabolic relationships, whereas dashed arrows indicate indirect metabolic relationships. Yellow ellipses represent volatile metabolites.

Differential non-volatile metabolites were enriched in pathways including vitamin digestion and absorption, the pentose phosphate pathway, amino sugar and nucleotide sugar metabolism, fat digestion and absorption, purine metabolism, regulation of lipolysis in adipocytes, glycerolipid metabolism, and thermogenesis (Figure 4a). In contrast, pathway enrichment of differential volatile metabolites identified only two pathways: inflammatory mediator regulation of TRP channels and metabolic pathways (Figure 4b).

Previous sensory and volatile studies provide a useful context for interpreting the present sex effects. In *E. chloroticus*, trained-panel evaluation characterized testes as sweeter and dairy-like and ovaries as more bitter, sour, herbaceous, and metallic [20]. The greater sweetness response of male *H. crassispina* gonads is broadly consistent with these observations, whereas their stronger E-tongue bitterness indicates that the direction of sex-related taste differences may depend on species, reproductive stage, and analytical approach. GC–MS/olfactometry studies of *M. nudus* further showed that gonad aroma reflects a balance between pleasant ester- and aromatic aldehyde-related notes and undesirable low-threshold sulfur compounds [23,48,53]. More directly, combined sensory and volatile profiling of *P. lividus* identified sex as a major source of variation [54], while recent volatilomics reported greater abundances of low-threshold aldehydes, alcohols, and D-limonene in females but more branched hydrocarbons and long-chain alcohols in males [55]. The female-biased terpenoids and male-biased hydrocarbons observed here partly agree with these patterns, whereas the male enrichment and high rOAV of 3-carene suggest species-specific odor markers. Although metabolomics under ocean warming and acidification has also revealed sex-dependent changes in sensory-related gonadal metabolites [56], intrinsic differences under normal conditions remain insufficiently resolved. By jointly relating electronic sensory fingerprints to non-volatile and volatile metabolomes, the present study extends previous targeted or stress-based investigations and offers an integrated biochemical framework for sex-dependent gonad flavor in *H. crassispina*.

Against this background, the integrated results support three principal associations rather than confirmed causal mechanisms (Figure 4c). First, differences in amino acid-, dipeptide-, and nucleotide-related metabolites coincided with the separation of female and male E-tongue fingerprints. Second, sugar phosphates, amino sugars, and vitamin/coenzyme-related metabolites contributed to the metabolic distinction between sexes, although their direct sensory relevance remains unclear. Third, differences in lipid-related metabolites occurred together with distinct volatile profiles and E-nose responses. These associations do not establish metabolic flux, precursor–product relationships, or causal contributions of individual metabolites to sensory perception.

Overall, female gonads were characterized by higher protein content, higher relative abundances of several phospholipids and nucleotide/coenzyme-related metabolites, and stronger umami and salty/umami E-tongue responses. Male gonads showed higher relative abundances of several acylcarnitines and hydroxy- or oxidized-fatty-acid-related metabolites, together with stronger sweetness and bitterness responses. The sexes also differed in specific E-nose responses and volatile compound classes, with 3-carene identified as a candidate rather than a confirmed odor-active marker in male gonads. However, the relatively small number of biological replicates used for metabolomic and sensory analyses (*n* = 3 per sex) should be considered a limitation of this study. Further studies with larger sample sizes and targeted validation are needed to confirm the contribution of key metabolites to sex-dependent flavor differences.

## 4. Conclusions

This study integrated basic quality assessment, E-tongue, E-nose, LC-MS, and HS-SPME-GC-MS to compare quality and flavor differences between female and male *H. crassispina* gonads. No significant sex differences were observed in wet weight, test diameter, GSI, moisture, or crude fat. However, female gonads had higher crude protein content, lightness, and yellowness, with an overall color closer to the preferred yellow to orange-yellow appearance of high-quality sea urchin gonads.

E-tongue and E-nose analyses revealed sex-dependent taste and odor fingerprints. Female gonads showed stronger umami, salty/umami, and sourness responses, whereas male gonads showed stronger sweetness, bitterness, and comprehensive aftertaste responses. Female gonads also showed higher W5S, W6S, and W1W responses, while male gonads showed higher W3C, W1S, and W2S responses. Metabolomics indicated that these flavor differences were mainly associated with differential accumulation of lipids and lipid-like molecules, organic acids and derivatives, dipeptides, nucleotide precursors, carbohydrate metabolism-related compounds, and volatile compounds, including terpenoids, hydrocarbons, aldehydes, ketones, and esters. Phospholipids, lysophospholipids, dipeptides, and nucleotide-related compounds were enriched in female gonads, whereas acylcarnitines, hydroxy fatty acids, 3-carene, and some hydrocarbons were more abundant in male gonads.

Integrated pathway analysis suggested that sex may shape taste and odor formation by regulating amino acid and peptide metabolism, nucleotide metabolism, carbohydrate metabolism, vitamin/coenzyme metabolism, lipid storage, and fatty acid oxidation. These findings provide metabolomic evidence for sex-dependent flavor formation in sea urchin gonads and support quality evaluation and refined utilization of sea urchin products. Future studies combining targeted quantification, GC-O, and human sensory evaluation are needed to validate the actual contributions of key flavor compounds.

## Figures and Tables

**Table 1:** Basic biometric parameters, gonadosomatic index (GSI), and proximate composition of male and female *Heliocidaris crassispina*.

	Male (*n* = 12)	Female (*n* = 8)	*p* Value
Wet weight (g)	62.61 ± 21.12	70.89 ± 20.92	0.400
Test diameter (mm)	50.16 ± 4.10	52.63 ± 6.80	0.322
GSI (%)	5.88 ± 2.20	5.98 ± 1.14	0.912
Moisture (%)	67.09 ± 0.55	67.64 ± 0.64	0.321
Crude ash (%)	8.90 ± 0.72	5.14 ± 0.22	0.001
Crude fat (%)	29.73 ± 0.25	29.51 ± 0.98	0.730
Crude protein (%)	36.18 ± 0.05	42.97 ± 0.07	<0.001

Moisture content is expressed on a wet weight basis, whereas crude protein, crude fat, and ash are expressed on a dry weight basis. Data are presented as mean ± SD. *p*-values were calculated using independent-sample *t*-tests.

**Table 2:** Color characteristics of male and female *Heliocidaris crassispina*.

	Male (*n* = 12)	Female (*n* = 8)	*p*-Value
L*	48.34 ± 1.65	57.19 ± 0.85	<0.001
a*	20.35 ± 1.30	17.88 ± 2.12	0.006
b*	32.93 ± 1.26	43.78 ± 2.25	<0.001
Δ*E*_1_	35.33 ± 2.02	23.21 ± 1.08	<0.001
Δ*E*_2_	43.14 ± 2.01	30.43 ± 1.33	<0.001

Data are presented as mean ± SD. *p*-values were calculated using independent-sample *t*-tests.

## Data Availability

The original contributions presented in this study are included in the article/Supplementary Materials. Further inquiries can be directed to the corresponding author.

## Supplementary Materials

The following supporting information can be downloaded at: https://www.mdpi.com/article/10.3390/foods15162780/s1, Table S1: List of Classification table of 3660 non-volatile metabolites; Table S2: Classification table of 366 significantly differentially non-volatile metabolites; Table S3: Correlation analysis between differential non-volatile metabolites and electronic tongue responses; Table S4: List of Classification table of 621 volatile metabolites; Table S5: Classification table of 53 significantly differentially volatile metabolites; Table S6: Correlation analysis between differential volatile compounds and electronic nose responses.
